# New Exercise-Dipyridamole Combined Test for Nuclear Cardiology in Insufficient Effort: Appropriate Diagnostic Sensitivity Keeping Exercise Prognosis

**DOI:** 10.5935/abc.20150051

**Published:** 2015-08

**Authors:** Inés Vidal Cortinas, Mario Beretta, Omar Alonso, Fernando Mut

**Affiliations:** Departamento de Medicina Nuclear do Hospital ‘Asociación Española’, Br. Artigas 1515, Montevideo – Uruguay

**Keywords:** Coronary Artery Disease, Exercise Test, Myocardial Perfusion Imaging / methods, Dipyridamole / diagnostic use

## Abstract

**Background:**

Myocardial perfusion scintigraphy (MPS) in patients not reaching 85% of the maximum predicted heart rate (MPHR) has reduced sensitivity.

**Objectives:**

In an attempt to maintain diagnostic sensitivity without losing functional exercise data, a new exercise and dipyridamole combined protocol (EDCP) was developed. Our aim was to evaluate the feasibility and safety of this protocol and to compare its diagnostic sensitivity against standard exercise and dipyridamole protocols.

**Methods:**

In patients not reaching a sufficient exercise (SE) test and with no contraindications, 0.56 mg/kg of dipyridamole were IV administered over 1 minute simultaneously with exercise, followed by 99mTc-MIBI injection.

**Results:**

Of 155 patients, 41 had MPS with EDCP, 47 had a SE test (≥ 85% MPHR) and 67 underwent the dipyridamole alone test (DIP). They all underwent coronary angiography within 3 months. The three stress methods for diagnosis of coronary lesions had their sensitivity compared. For stenosis ≥ 70%, EDCP yielded 97% sensitivity, SE 90% and DIP 95% (p = 0.43). For lesions ≥ 50%, the sensitivities were 94%, 88% and 95%, respectively (p = 0.35). Side effects of EDCP were present in only 12% of the patients, significantly less than with DIP (p < 0.001).

**Conclusions:**

The proposed combined protocol is a valid and safe method that yields adequate diagnostic sensitivity, keeping exercise prognostic information in patients unable to reach target heart rate, with fewer side effects than the DIP.

## Introduction

The reduced diagnostic sensitivity of myocardial perfusion scintigraphy (MPS) in patients not achieving a sufficient exercise (SE) test is well recognized^1-4^. Many laboratories, in the absence of contraindications, usually perform a vasodilator test in these patients, either on the same session or on a separate one. However, this procedure is time consuming and cost-inefficient, since the stress laboratory personnel needs to perform and monitor a full second stress test.

Knowing the functional capacity is important for prognosis in patients evaluated for coronary artery disease (CAD)^5-9^. The same can be said of the chronotropic response to exercise and the speed of heart rate recovery^10,11^. Therefore, in order to improve diagnostic sensitivity without losing functional ergometric data, patients with insufficient exercise tests could benefit from a combined protocol that uses vasodilators in addition to physical stress. Another advantage would be the fact that patients would not be transferred from the treadmill or bicycle to a stretcher, where they would wait for recovery before receiving the pharmacologic stimulus.

Adequate diagnostic sensitivity has been reported in patients with insufficient exercise tests and limited exercise capacity by using a combined protocol of exercise and dipyridamole^12^. Also, the side effects of dipyridamole are less serious in patients who exercise at the same time^13-16^. Further consideration must be given to the improved diagnostic quality of the scintigraphic images achieved with the simultaneous injection of the vasodilator. This is due to a diminished hepatic and intestinal uptake of the radiotracer, which is usually high after dipyridamole^13-16^.

**The objectives of our investigation were:**

1. To establish the safety and sensitivity for CAD diagnosis of a new protocol combining exercise and dipyridamole given over the course of 1 minute;
  To validate that protocol use for patients performing insufficient exercise test by comparing its diagnostic sensitivity with those of conventional tests: SE test and dipyridamole alone test (DIP).

## Methods

**Figure 1:**
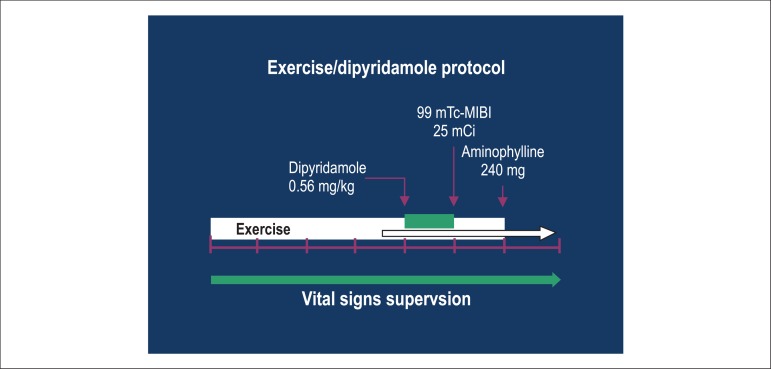
Schematic representation of the proposed combined protocol

Since March 2004, every patient submitted to stress MPS, who performed an insufficient exercise test [who did not reach at least 85% of the maximum predicted heart rate (MPHR) for their age, calculated as 220 - age in years] and had no contraindication for vasodilators, was injected with 0.56 mg/kg of dipyridamole over 1 minute while maintaining the physical stress. One minute after the completion of dipyridamole infusion, ^99m^Tc-methoxy-isobutyl-isonitrile (^99m^Tc-MIBI) was intravenously (IV) administered at the dose of 14 MBq/kg. If the patient was unable to keep exercising at the maximum effort achieved, the workload was lowered during the dipyridamole infusion. This allowed all patients to keep at least some level of exercise until test completion. One minute after the tracer injection, 240 mg of aminophylline were IV administered, and upon completion, exercise was stopped (Figure 1).

Due to the incorporation of this protocol, all patients were instructed to fast for at least 2 hours prior to the test and to abstain from caffeine and other xanthines for at least 24 hours. Informed consent was obtained in all cases. Exercise was performed on a cycle-ergometer placed vertically, adding weights of 150 or 300 kgm/min at each stage.

The protocol included the acquisition of a post-stress gated SPECT scan, approximately 30-45 minutes after the injection of the radiotracer, and of a rest gated SPECT on a separate day. Studies were obtained using a dual-head camera equipped with high-resolution collimators, 180° rotation, 32 projections, 40 s/projection, 8 frames/cycle, no arrhythmia rejection and a 64×64 matrix with 1.5 zoom. These studies were reconstructed using iterative algorithm (OSEM) without attenuation or scatter correction, and realigned along the heart axis. Image interpretation was performed by one or more members of our medical staff. Diagnostic criteria were based on the presence of reversible perfusion defects (considered as ischemia), fixed perfusion defects (considered as infarction), or partially reversible defects (considered as infarction plus ischemia). No quantitative analysis was used, but rather the visual analysis of experienced observers.

We included patients studied with MPS using our exercise-dipyridamole combined protocol (EDCP), patients having SE tests, and patients undergoing conventional DIP (0.56 mg/kg over 4 minutes). All patients included in this investigation were studied during the first half of 2012, and the selection criterion was that they should have a coronary angiography performed no later than 3 months after the MPS.

Side effects of the new protocol were recorded and compared with those of the conventional DIP.

The diagnostic sensitivity of the proposed protocol for severe coronary lesions (stenosis of ≥ 70%, and ≥ 50% in case of left main coronary artery involvement) as well as for moderate ones (stenosis of ≥ 50%) was determined. Sensitivity was calculated with the 95% confidence interval (95% CI). The Student’s *t* Test was used for the comparison of sample means, and the Chi‑square Test was used for categorical data comparison among different subsamples. Group differences were considered significant at p < 0.05, and calculations were performed using GraphPad Prism software, version 6.00 for Windows (GraphPad Software, La Jolla, California, USA).

## Results

Of 155 patients recruited, the EDCP was followed in 41 [73% male, mean age 62.26 (SD = 9.4) years]. Forty-seven patients [72% male, mean age 59.8 (SD = 9.2) years] underwent a SE test, while the DIP was performed in 67 patients [51% male, mean age 64 (SD = 9.2) years]. There were no significant differences in the mean age of the three groups of patients. Risk factors resulted similar among groups, except for overweight, which was less prevalent in the SE group (Table 1). There were no significant differences regarding the presence of previous myocardial infarction: 15 patients in the EDCP group; 14 patients in the SE group; and 22 patients in the DIP group (p = 0.79).

**Table 1:** Risk factors in the three groups of patients

**Risk factor**	**EDCP (n = 41)**	**SE (n = 47)**	**DIP (n = 67)**	**p value**
Diabetes	13 (32%)	9 (19%)	19 (28%)	0.36
Hypertension	27 (66%)	31 (66%)	52 (78%)	0.28
Dyslipidemia	26 (63%)	27 (57%)	35 (52%)	0.52
Smoking	12 (29%)	9 (19%)	17 (25%)	0.53
BMI ≥ 25	25 (61%)	16 (34%)	40 (60%)	0.01
Family history	15 (37%)	24 (51%)	34 (51%)	0.29

EDCP: Exercise-Dipyridamole Combined Protocol; SE: Sufficient exercise test; DIP: Dipyridamole only; BMI: body mass index.

Patients undergoing SE test reached a higher mean heart rate and developed a higher metabolic output than those following the combined protocol: 87% vs. 70% of MPHR, and 5.8 (SD = 1.56) vs. 5.1 (SD = 1.57) METs, respectively, each with p = 0.03. In patients undergoing EDCP, mean systolic blood pressure was 180 (SD = 27) mm Hg at maximum effort and decreased to 152 (SD = 24) mm Hg after dipyridamole injection (p < 0.001), while mean diastolic blood pressure values were 103 (SD = 13) mm Hg and 88 (SD = 13) mm Hg, respectively (p < 0.001).

**Figure 2:**
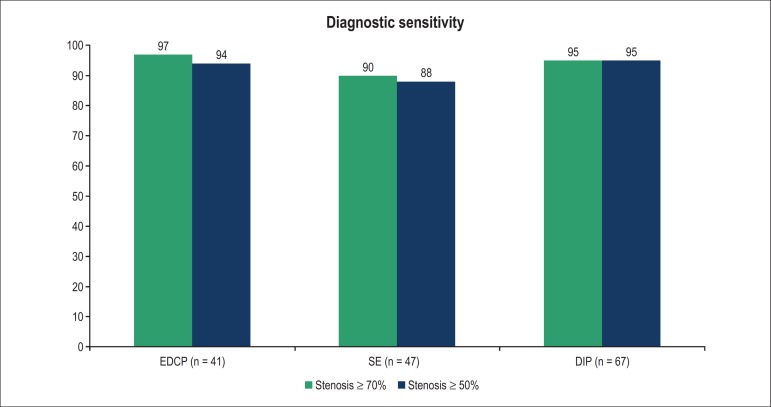
Comparison of diagnostic sensitivity of the three protocols for coronary stenosis ≥ 70% and ≥ 50%.

EDCP: Exercise-Dipyridamole Combined Protocol; SE: Sufficient exercise test; DIP: Dipyridamole only; V: Vessel

There were no significant differences in CAD prevalence among the three groups of patients, either regarding the severity of the lesions or the number of affected vessels (Table 2). In 83% of the patients, severe coronary lesions were found (76% of those following the EDCP, 83% with SE, and 87% with DIP). In 42%, one vessel was affected, in 24%, two vessels, and in 17%, three vessels or its equivalent (left main and right coronary artery). The combined protocol had 97% diagnostic sensitivity for severe coronary lesions (95% CI: 83.2% to 99.5%), while the SE test showed 90% (95% CI: 75.8% to 97.1%) and the DIP protocol, 95% (95% CI: 85.8% to 98.9%), with no significant difference among them (p = 0.43) (Figure 2). At least moderate coronary lesions were present in 88% of the total population (85% of patients following the EDCP, 87% of those with SE test, and 91% of those with DIP). The EDCP showed 94% sensitivity for moderate coronary lesions (95% CI: 80.8% to 99.1%); the ES test showed 88% (95% CI: 73.8% to 95.9%), and the DIP protocol, 95% (95% CI: 86.3% to 98.9%). These differences in sensitivity between the different types of protocols showed no statistical significance (p = 0.35) (Figure 2).

**Table 2:** Prevalence of CAD among the three groups

**Angiography**	**EDCP (n = 41)**	**SE (n = 47)**	**DIP (n = 67)**	**Total (n = 155)**	**p value**
Lesions ≥ 70%	31(76%)	39(83%)	58(87%)	128(83%)	0.45
3V	8(20%)	6(15%)	12(18%)	26(17%)	0.66
2V	8(20%)	10(21%)	19(28%)	37(24%)	0.51
1V	15(37%)	23(49%)	27(40%)	65(42%)	0.47
Lesions ≥ 50%	35(85%)	41(87%)	61(91%)	137(88%)	0.19
3V	12(29%)	11(23%)	15(22%)	38(25%)	0.71
2V	9(22%)	8(17%)	18(27%)	35(23%)	0.46
1V	14(34%)	22(47%)	28(42%)	64(41%)	0.48

EDCP: Exercise-Dipyridamole Combined Protocol; SE: Sufficient exercise test; DIP: Dipyridamole only; V: Vessel.

Regarding side effects, 49 patients in the DIP group (73%) showed one or more. Headache was present in 40% of the patients, flushing in 30%, weakness in 22%, gastric discomfort in 10%, and dizziness in 6%. Conversely, dizziness was the only side effect present with the EDCP (5 patients), usually in connection to a drop in blood pressure. These results establish a difference of statistical significance between both groups (p < 0.001). Dizziness was present in 5 patients (12%) of the EDCP and in 4 patients of the DIP group (6%) (p = 0.25). A mean drop of 26 (SD = 13.6) mm Hg in systolic blood pressure was found in patients who did not experience symptoms, while the decrease was 37 (SD = 15) mm Hg in patients with dizziness (p = 0.14).

No change in the PR interval was found with either the DIP protocol or the EDCP.

## Discussion

The idea of combining an insufficient exercise test with a dipyridamole stimulus in order to reach adequate diagnostic sensitivity without losing functional capacity data seems to be theoretically correct and to have achieved positive practical results. Candell-Riera et al.^12^ have shown that the diagnostic sensitivity for 50% coronary stenosis of their combined protocol (89%) was significantly higher than that of the insufficient effort test (71%) and comparable to that of the SE test (93%). We achieved similar results: the difference between the diagnostic sensitivity of our combined protocol and that of the SE for coronary lesions ≥ 50% was not significant. We also compared it to the DIP protocol alone and found no diagnostic difference either. However, we made no comparison with the diagnostic sensitivity of the insufficient exercise test alone. As a rule, no insufficient effort scintigraphy is performed in our laboratory except when it is specifically required by the attending physician. Before the EDCP was adopted, any patient who failed to reach at least 85% of the MPHR was laid in a stretcher and, after 10-15 minutes, underwent conventional dipyridamole test. With EDCP, adequate diagnostic sensitivity is achieved without losing information from the exercise test about functional capacity and chronotropic response. Furthermore, the total time required for completion of the full procedure is shortened.

Candell-Riera et al.^12^ have administered IV 0.56 mg/kg of dipyridamole over 4 minutes during the exercise test. The major differences with our protocol are that we give the dose in only 1 minute and that it is used in all patients not reaching sufficient effort, not only those achieving less than 5 METs. We chose to shorten the infusion time because most patients with insufficient exercise tests showed difficulties in maintaining the effort further. Unlike the investigation by the abovementioned authors, heart rate alone was taken into account and not METs; whenever 85% of MPHR was not reached – and no contraindications were present – dipyridamole was always administered. Our results seem to validate the followed criteria, since the diagnostic sensitivity of the EDCP was not significantly different from that of the SE test, even though some patients with a mean metabolic output higher than 5 METs (5.1, SD = 1.57) were included.

It could be argued that full vasodilation effect of dipyridamole might not have been reached in every case at the time of the radiotracer injection due to the short time allowed for the pharmacologic action. However, no difference in sensitivity was demonstrated compared to conventional protocols. This fact permits the assumption that vasodilation was adequate enough for diagnostic purposes. Furthermore, even if pharmacologic vasodilation was not optimal, it should be taken into account that some vasodilation was already present with exercise, and that myocardial extraction of the radiotracer is not linear with flow, especially at high values^17^, so a marginal increase with time might not be reflected in imaging.

We also confirmed the previously demonstrated fact that the combination of exercise with dipyridamole produces fewer side effects than the use of dipyridamole alone. Patients studied with the new combined protocol only presented dizziness in 12%, which can be associated with a decrease in blood pressure. This decline (rapidly compensated laying the patient down) could be explained by the vasodilatation effect of dipyridamole, as well as by the physiologic decrease resulting from exercise cessation or reduction. In fact, patients presenting dizziness underwent a more pronounced yet non-significant decrease in systolic blood pressure. Conversely, most patients (73%) studied with the DIP protocol experienced some kind of discomfort, mostly headache. The usual supine position during conventional pharmacologic stress allows better tolerance to hypotension than the upright position. Nevertheless, we did not find a statistical significant difference regarding the presence of dizziness for both groups.

At the beginning of the implementation of the combined protocol, we usually asked the patient to stop exercising after MIBI injection. Afterwards, aminophylline administration was started. We frequently observed a sudden drop in blood pressure, caused by the vasodilatation effect of dipyridamole added to the abrupt cessation of the physical effort. This was resolved by quickly positioning the patient in the decubitus position. In consequence, we decided thereafter to ask the patients to keep pedaling at a lower level until the aminophylline infusion was completed. As a result, the prevalence of dizziness as a side effect was reduced significantly, as described in the study.

The association of exercise with dipyridamole has also proved to increase image quality^13-16^. Even though image characteristics in the various protocols considered in our investigation were not specifically evaluated, all the images were adequate for diagnostic purposes according to standard criteria of interpretation.

To our knowledge, with the exception of our protocol and of that described by Candell-Riera et al.^12^, all others combining exercise with dipyridamole start first with the vasodilator drug, being then complemented with exercise, either at a low‑level or limited by symptoms^13,16,18^. However, this sequence does not allow clinical monitoring, which is more relevant in patients with exercise-related symptoms, or proper evaluation of functional capacity.

Ahlberg et al.^18^ reported that almost one third of patients referred for MPS reached 85% of their MPHR. If this had been known in advance, the stimulation with dipyridamole before the exercise would have been unnecessary. These authors proved the prognostic value of their protocol combining dipyridamole with symptom-limited exercise, but they recognized limitations by not comparing with a similar group of patients reaching suboptimal effort or using dipyridamole alone. An editorial^19^ written about their work mentions the need of a control group, such as dipyridamole with low-level exercise, dipyridamole alone, or exercise alone. In addition, the lack of coronary angiography to evaluate the sensitivity and specificity of the test is highlighted. Therefore, it is not known whether the dipyridamole/exercise test limited by symptoms improves the diagnostic sensitivity for CAD when compared to other protocols. The same conclusions could be drawn from the analysis of adenosine/exercise protocols.

An approach similar to ours has been possible with regadenoson, a vasodilating drug, which specifically stimulates the A2a receptors. In recent works by Parker et al.^20^ and Ross et al.^21^, regadenoson was administered at peak exercise in bolus injection if the patient had not reached 85% of the MPHR. However, the subjects studied were submitted to a pharmacologic stress and agreed to undergo an exercise test as an additional procedure. They were not patients directly assigned to an exercise MPS who did not reach the target heart rate. Parker et al.^20^ reported that 50% of their patients were able to reach 85% of the target heart rate, while this was the case in 62.5% of the patients in the series by Ross et al.^21^. Hence, at least half of their patients did not formally require the administration of a vasodilator stimulus since the exercise was adequate. This highlights a frequently mistaken medical perception of the patient’s exercise capacity when perfusion tests are indicated. It also supports the design of a protocol geared towards complementing an insufficient exercise test while at the same time saving the prognostic functional data of physical stress.

Therefore, every patient referred to our department for an exercise MPS is instructed to abstain from xanthines for 24 hours previous to the test, just in case dipyridamole has to be administered in a combined protocol. In regard to this, we share the idea of administering a vasodilator when necessary^22^. Both Parker et al.^20^ and Ross et al.^21^ have demonstrated that their respective protocols are feasible and safe, and are associated with fewer side effects compared to the administration of regadenoson only. However, their patients underwent no coronary angiography, so the diagnostic sensitivity was not determined.

The current work proves that our EDCP is feasible, safe, and, more importantly, has a diagnostic sensitivity at least similar to that provided by a SE test or a DIP test alone. Together with the evaluation of exercise-induced symptoms, ECG changes, functional capacity and chronotropic response, we could say that, paraphrasing Hendel and Frost^22^, with our combined protocol, we work off-label, on target and with diagnostic accuracy.

### Limitations

The coronary anatomy of only a few dozens of the hundreds of patients undergoing the EDCP was established, because the indication of a coronary angiography depended mainly on a positive nuclear test result, producing a strong referral bias that explains the high prevalence of CAD. This was an obvious obstacle to evaluate specificity, accuracy, as well as positive and negative predictive values of the technique.

## Conclusions

We demonstrated that our combined protocol for MPS is well tolerated and yields at least similar diagnostic sensitivity as compared to a SE test or a DIP test alone. It preserves prognostic information from the exercise even when the test is insufficient. Additionally, it can be safely completed in a shorter time than other proposed combined protocols with dipyridamole, and close to that using regadenoson.
